# Chitosan-Coated 5-Fluorouracil Incorporated Emulsions as Transdermal Drug Delivery Matrices

**DOI:** 10.3390/polym13193345

**Published:** 2021-09-29

**Authors:** Taif Ali Khan, Abul Kalam Azad, Shivkanya Fuloria, Asif Nawaz, Vetriselvan Subramaniyan, Muhammad Akhlaq, Muhammad Safdar, Kathiresan V. Sathasivam, Mahendran Sekar, Omji Porwal, Dhanalekshmi Unnikrishnan Meenakshi, Rishabha Malviya, Mireia Mallandrich Miret, Ajay Mendiratta, Neeraj Kumar Fuloria

**Affiliations:** 1 Faculty of Pharmacy, Gomal University, Dera Ismail Khan 29050, Pakistan; 01drazad@gmail.com (T.A.K.); asifnawaz676@gmail.com (A.N.); dr.akhlaq@gu.edu.pk (M.A.); safdarlaghari10@gmail.com (M.S.); 2 Advanced Drug Delivery Laboratory, Faculty of Pharmacy, International Islamic University Malaysia, Kuantan 25200, Pahang, Malaysia; aphdukm@gmail.com; 3 Faculty of Pharmacy, AIMST University, Bedong 08100, Kedah, Malaysia; 4 Centre of Excellence for Biomaterials and Engineering, AIMST University, Bedong 08100, Kedah, Malaysia; skathir@aimst.edu.my; 5 Faculty of Medicine, Bioscience and Nursing, MAHSA University, Jalan SP 2, Bandar Saujana Putra, Jenjarom 42610, Selangor, Malaysia; drvetriselvan@mahsa.edu.my; 6 Faculty of Applied Science, AIMST University, Bedong 08100, Kedah, Malaysia; 7 Department of Pharmaceutical Chemistry, Faculty of Pharmacy and Health Sciences, Universiti Kuala Lumpur Royal College of Medicine Perak, Ipoh 30450, Perak, Malaysia; mahendransekar@unikl.edu.my; 8 Department of Pharmacognosy, Tishk International University, Erbil 44001, KRG, Iraq; omji.porwal@tiu.edu.iq; 9 College of Pharmacy, National University of Science and Technology, Muscat 130, Oman; dhanalekshmi@nu.edu.om; 10 Department of Pharmacy, SMAS, Galgotias University, Gautam Buddh Nagar, Greater Noida 201310, India; rishabha.malviya@galgotiasuniversity.edu.in (R.M.); ajay.20smas3020002@galgotiasuniversity.edu.in (A.M.); 11 Department of Pharmacy, Pharmaceutical Technology and Physical-Chemistry, Faculty of Pharmacy and Food Sciences, University of Barcelona, 08028 Barcelona, Spain; mireia.mallandrich@ub.edu

**Keywords:** chitosan, 5-fluorouracil, surfactants, emulsions, transdermal delivery

## Abstract

The purpose of the present study was to develop emulsions encapsulated by chitosan on the outer surface of a nano droplet containing 5-fluorouracil (5-FU) as a model drug. The emulsions were characterized in terms of size, pH and viscosity and were evaluated for their physicochemical properties such as drug release and skin permeation in vitro. The emulsions containing tween 80 (T80), sodium lauryl sulfate, span 20, and a combination of polyethylene glycol (PEG) and T20 exhibited a release of 88%, 86%, 90% and 92%, respectively. Chitosan-modified emulsions considerably controlled the release of 5-FU compared to a 5-FU solution (*p* < 0.05). All the formulations enabled transportation of 5-FU through a rat’s skin. The combination (T80, PEG) formulation showed a good penetration profile. Different surfactants showed variable degrees of skin drug retention. The ATR-FTIR spectrograms revealed that the emulsions mainly affected the fluidization of lipids and proteins of the stratum corneum (SC) that lead to enhanced drug permeation and retention across the skin. The present study concludes that the emulsions containing a combination of surfactants (Tween) and a co-surfactant (PEG) exhibited the best penetration profile, prevented the premature release of drugs from the nano droplet, enhanced the permeation and the retention of the drug across the skin and had great potential for transdermal drug delivery. Therefore, chitosan-coated 5-FU emulsions represent an excellent possibility to deliver a model drug as a transdermal delivery system.

## 1. Introduction

The transdermal drug delivery system (TDDS) offers several advantages over the other conventional drug delivery systems [1]. The TDDS is known to avoid the first-pass metabolism, offer stable drug delivery, exhibit a decreased systemic drug interaction, improve patient compliance, reduce the frequency of drug administration, and offer higher therapeutic efficacy and safety [2,3]. The infiltration of drugs into skin and their flexible expansion are constrained by the obstacle function of the highly structured nature of the stratum corneum (SC) components [4]. Various permeation enhancers have been used to enhance the topical delivery of drugs, i.e., dimethyl sulfoxide (DMSO), dimethylacetamide (DMAC), dimethylformamide (DMF) [5,6], pyrrolidone’s [7], cyclodextrins [8], and azones [9]. However, permeation enhancers are associated with various problems, for example, an increase in the concentration of DMSO can trigger erythema or SC swelling. It can also cause the denaturing of skin proteins that may lead to scaling, stinging, erythema, irreversible membrane damage, urticaria contact, and a burning sensation as well [10,11].

Chitosan is one of the commonly used polymers in transdermal drug delivery systems due to the fact that it is biocompatible, non-toxic and improves the drug absorption through the skin epithelial layers [12]. It helps to increase the permeation of hydrophobic drugs through the skin and drug retention in the dermal epidermis via an interaction with the skin surface that leads to a change in the SC morphology and the disruption of the tight junctions of the corneocyte layers [13].

The strong interaction of chitosan with the skin surface allows for a long retention time and the enhancement of the permeation/absorption of drugs across the skin [14,15]. This can be attributed to the dual combination factors, such as: (1) mucoadhesive properties and (2) the transient opening of cellular tight junctions for the passage of hydrophilic macromolecules [16,17]. Chitosan improves the permeability of 5-fluorouracil (5-FU) across SC by transforming the prearrangement of phospholipids in the epithelial cell membrane that enhances the fluidity of lipid bilayers in the skin membrane. As a result, it may lead to the transportation of 5-FU via the transcellular pathway. 5-FU is a highly polar drug molecule that has been commonly prescribed for cancer treatment since the 1930s. However, in the USA the topical use of 5-FU for superficial cancer lesions was approved in the 1970s [18].

Attributed to low drug permeation through the SC barrier, the conventional topical formulation is limited to superficial dermal layers and requires a 5-FU dose of about 5% to achieve the desired drug effect. Hence, there is a high risk of undesired side effects and toxicity that may cause poor patient adherence to such treatment [19]. Some recently approved commercial topical formulations of 5-FU (0.5%) include Carac^®^; Sanofi, Gentilly, France [20], Fluoroplex 1% 5-FU solution (Allergan, Inc., Irvine, CA, USA), creams (Efudex^®^, Valeant Pharmaceuticals, Bridgewater, NJ, USA) and a 0.5% microsphere-based cream (Carac^®^, Valeant Pharmaceuticals) [21]. A report has suggested that commercially available topical products offer the demerit of low retention time at the delivery site that results in inadequate skin permeation along with skin irritation reactions, such as dryness, redness, swelling, and burning pain of the upper layer of skin [22,23].

To overcome the problem of inadequate skin permeation, the incorporation of 5-FU into the transdermal drug delivery system using emulsions may enhance the 5-FU permeation effectively into the deeper layers of skin with fewer adverse effects. Therefore, in the present study chitosan was used as a coating material to prepare emulsions of 5-fluorouracil. The formulated emulsions of 5-FU were further investigated for the influence of various surfactants on their physicochemical properties. Moreover, this study also involved the addition of diverse surfactants. For examples, span 20, SLS, T80, and PEG 4000, that were investigated in terms of their influence on the physicochemical characteristics that have potential effects on drug release and skin permeation of 5-FU across the skin.

The present study highlights the formulation of modified chitosan- (α-type chitosan) coated 5-FU emulsions for the first time, with enhanced permeability and retention across the skin. The findings of the present study will support the scientific community in the development of emulsions of 5-FU as a transdermal drug delivery system to deliver the model drug efficiently for cancer treatment. This study is not only expected to offer better drug delivery options in comparison to conventional drug therapies, but it also shows how the dosing related side effects and toxicity can be overcome.

## 2. Materials and Methods

### 2.1. Materials

5-Fluorouracil was obtained from Biolabs Pharma Pvt. Ltd., (Biolabs Pharma, Islamabad 45700, Pakistan). Olive oil was purchased from Hamdard Laboratories (Hamdard Laboratories, Karachi 76400, Pakistan). The sodium lauryl sulfate, Tween 80 and 20, chitosan derivatives and span 20 were procured from Merck (Merck KGaA, D-64293 Darmstadt, Germany) and Polyethylene glycol was obtained from Olive laboratories (Olive laboratories, Rawalpindi 46600, Pakistan).

### 2.2. Preparation of the Emulsions

Oil in water (O/W) emulsions were formulated by mixing different ratios of oil, surfactant, and aqueous solutions. The aqueous phase was prepared by dissolving the derivatives of chitosan (0.25 g *w*/*v*) in distilled water (44.4 mL *w*/*v*), followed by a drop wise addition to olive oil (5 mL *w*/*v*) and 0.25 g w/v of T80, SLS, Span 20 and PEG, respectively. The mixture was continuously homogenized for 2 min at 10000 rpm using a homogenizer (Daihan Scientific Co. Ltd., SANGWOLGOG, ONG SUNGBUK KU, Seoul 136120, Korea). The prepared emulsions were finally stored at 25 °C for further experiments.

### 2.3. Physicochemical Characterization of the Emulsions

#### 2.3.1. Size and Zeta-Potential

The size and zeta-potential of the emulsions were determined using the Malvern Zetasizer Nano ZS90 (Malvern Instruments LTD., Malvern, Worcestershire, WR14 1AT, United Kingdom) as per the standard procedure with a minor modification [24]. Briefly, the test sample was diluted by ultrapure water (1:10) ratio. It was measured at a 90° angle using a disposable electrode cuvette after rinsing with ethanol and ultrapure water.

#### 2.3.2. Morphology of Emulsions

A light microscope (CX41RF, Olympus, Shinjuku-Ku, 163-0914 Tokyo, Japan) was used to observe the microscopic morphology of the emulsions. It was equipped with a digital eyepiece connected with a camera. A total of 1 mL of the emulsions was dropped on the glass slide and a thin smear was formed under the microscope to observe the shape and size of the emulsions.

#### 2.3.3. pH of the Emulsions

The pH of a dermal emulsion is an important factor to be considered for skin compatibility. The electrode of the pH meter was immersed in 10% of each emulsion to detect the pH [25].

#### 2.3.4. Viscosity Determination

A Brooke Field Viscometer (RVTD, Middleboro, Stoughton, 02072-MA, USA) equipped with an UN-adapter was used to measure the viscosity of the emulsions at a temperature of 25 °C. All the experiments were carried out in triplicate.

#### 2.3.5. Emulsification and Phase Separation Study

The emulsions were diluted in series and a change in phase was observed optically. Briefly, oil was added to the surfactants in series ranging from 1:1 to 1:9 and added to 50 mL of distilled water. It was kept for 2 h, then a UV reading was measured at 260 nm by a UV-vis spectrophotometer (Shimadzu 1601, Shimadzu, Kyoto 604-8511, Japan). In the phase separation study, 1 mL of the emulsions was taken into three different 10 mL volumetric flasks, and distilled water was added up to a mark to dilute it. It was inverted several times until a proper mixture was formed and stored for 2 h. The visual inspection was performed to determine the phase separation of the emulsions according to the previous method [26].

#### 2.3.6. Drug Loading

Different quantities of the drug in increasing order were dissolved in the emulsions. Next, centrifugation was performed at 5000 rpm for 30 min to collect the supernatant and then it was dissolved in a suitable solvent. The absorbance was measured at 260 nm (Shimadzu 1601, Shimadzu, Kyoto 604-8511, Japan) [27]. The % of drug entrapment efficacy and loading capacity were estimated according to Equation (1):

(1) $$Loading\;capacity\;\left(\%\right)=\frac{Quantity\;of\;drug\;added-Quantity\;of\;free\;drug}{weight\;of\;nanoemulsions}\times 100$$

#### 2.3.7. Stability Study of the Emulsions

To determine the stability of the emulsions, various temperatures (5 °C, 25 °C and 40 °C) were employed up to 30 days for a visual observation of any cracking, creaming or phase separation [28].

#### 2.3.8. Skin Irritancy Test

The skin irritancy test was performed based on the standard procedure using male Sprague Dawley rats [29]. A single dose (1 mL) of the emulsions was applied to the left ear (treatment) and right ear (control) of the rat and any development of erythema was noticed over a period of 24 h.

### 2.4. In Vitro Release of the Emulsions

The in vitro drug release profile of the emulsions was performed using Franz diffusion cells (K-C type, Pakistan). The donor and receiver chambers were separated by a cellophane membrane (pore size: 0.45 µm). For the study, 1 mL of the emulsion was placed on the surface of the prepared cellophane membrane. A phosphate buffer saline (PBS, pH 5.5) was utilized as a dissolution media. The temperature of the cells (32 ± 1 °C) was maintained by covering the water jacket (in simulation with the skin surface temperature) and the dissolution media was constantly stirred at 100 rpm. The samples (2 mL) were taken at each specific time interval (0, 0.5, 1, 2, 4, 8, 12, 16, 20 and 24 h) and diluted up to 5 mL with a substitute of the dissolution media. The samples were scanned at 260 nm by a UV spectrophotometer (Shimadzu 1601, Shimadzu, Kyoto 604-8511, Japan). Then, the percentage (%) of cumulative drug release was estimated [30,31].

Another method used to study the release of 5-FU from the emulsions was the centrifugation method. Briefly, in this method the PharmTest dissolution apparatus (Pharma Test Apparatebau AG, Siemensstrasse 5. D-63512 Hainburg, Germany) was used. A total of 5 mL of the emulsions was added into the USP apparatus II (Paddle) with 500 mL of release medium (Phosphate Buffer, pH 5.5). The temperature of the release medium was maintained at 32 ± 1 °C and the paddle rotation rate was maintained at 100 rpm. A total of 5 mL of the sample was taken at specific times intervals (0, 0.5, 1, 2, 4, 8, 12, 16, 20 and 24 h) and centrifuged at 1000× for 5 min. The filtrate was collected and analyzed on a UV spectrophotometer [32]. The dissolution profile of the 5-FU solution was obtained in a similar way.

### 2.5. Drug Release Kinetics

The Weibull equation was considered to determine the drug release kinetics in this study [33,34]. The obtained data were fitted according to Equation (2).

(2) $$\frac{M_{t}}{M_{\infty }}=1-e^{-at^{b}}$$

where, *M_t_* is the accrued mass dissolved at time *t*, *M_∞_* is the mass dissolved at an infinite time, *a* is the scale parameter and *b* is the shape parameter.

### 2.6. Ex Vivo Skin Permeation of the Drug

Franz diffusion cells were used to determine the skin permeation ability in ex vivo studies (K-C type, locally made, Pakistan) through freshly collected rat’s skin. The rat’s cervical part (Sprague Dawley; 200–250 g/kg/b.wt.) was separated by humane sacrificing. The abdomen section was marked and carefully shaved using a sharp razor blade. Excessive fat was removed from the subcutaneous parts of the entire abdomen skin using a surgical seizure. It was then gently washed with normal saline (0.9% NaCl) and stored at −20 °C by wrapping with aluminum foil for further use. The skin was pre-hydrated for 2 h to soften. Next, the SC (epidermis) side was placed facing the donor chamber while the dermal side was placed facing the receiver chamber. After that, they were carefully placed inside the Franz diffusion cell. After maintaining the temperature at 37 ± 1 °C and filling the receiver compartment with PBS (pH 7.4), it was stirred by a magnetic rotor at a speed of 100 rpm. After the donor compartment was filled with 1 mL of the emulsions, it was sealed with parafilm to maintain the occlusive conditions included in the samples. The samples (2 mL) were transferred to a tube at routine intervals (0, 0.5, 1, 2, 4, 8, 12, 16, 20 and 24 h). The obtained samples were filtered using a membrane filter (0.2 µm) and the absorbance was measured by a UV spectrophotometer [35].

### 2.7. Skin Drug Retention

Following the permeation test, phosphate buffer saline (PBS) with pH 7.4 was used to wash the skin to eliminate the additional formulation from the skin surface. Then the diffusion zone was slashed into a small section and dispersed in PBS (pH 7.4). It was then sonicated for 10 min and homogenized for 5 min. The homogenized sample was centrifuged for 15 min to collect the supernatant. Finally, it was filtered using a HPLC filter (0.2 µm) and the absorbance was measured at 260 nm (Shimadzu 1601, Shimadzu, Kyoto 604-8511, Japan) to determine the amount of skin drug retention.

### 2.8. Physicochemical Characterization of the Skin

The mechanism of the skin permeation of the emulsions was determined based on the physicochemical characterizations of the tested samples using ATR-FTIR chemical analysis. Following the drug permeation experiment, the skin was transferred and washed softly along with PBS (pH 7.4) to eliminate the emulsions from the skin surface. It was then placed on a zinc selenide crystal. An ATR-FTIR reading was taken with 16 cm^−1^ resolution, 675–4000 cm^−1^ and 1.5 min as the acquisition time [36].

### 2.9. Statistical Analysis

The obtained data were statistically analyzed using ANOVA (one-way analysis of variation) and *t*-test (IBM^®^ SPSS^®^ Statistics version 19, Armonk, New York 10504-1722, United States) and Statistical Package Minitab^®^ version 20 (Minitab, LLC, Pennsylvania, State College, PA 16801, USA). The data were statistically significant with a value of *p* ˂ 0.05. All the tested data were described as triplicate (n = 3) and mean ± standard deviation (S.D).

## 3. Results

### 3.1. Physicochemical Characterization of the Emulsions

The physicochemical properties provide a better insight into the formulation dynamics and a better understanding of the product concerning its application.

#### 3.1.1. Droplet Size and Zeta-Potential

**Figure 1:**
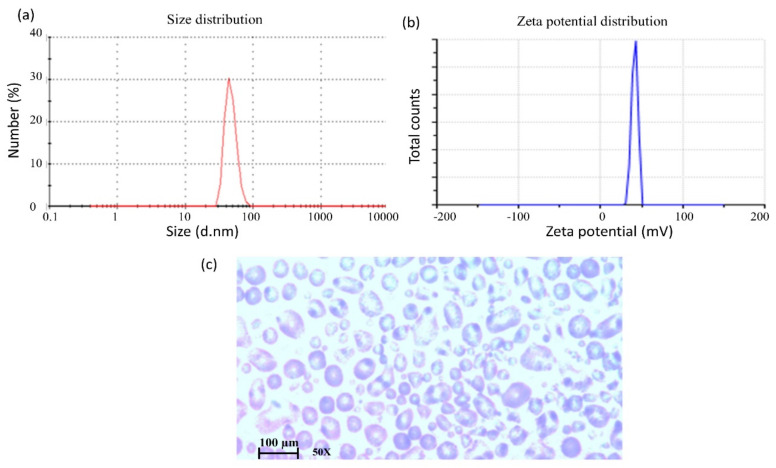
Representing the size (**a**) and zeta potential (**b**) of the emulsions and (**c**) A microscopic image of the emulsions containing 5-FU was captured using a light microscope (CX41RF, OLYMPUS), 50× magnification. Data were expressed as n = 3.

The overall particle size distribution indicates the quality of the formulation. The formulated emulsions exhibited a mean droplet size ranging from 109.6 ± 7.23 to 141.3 ± 9.31 nm in Table 1 with a narrow polydispersity index, which indicates a homogenous system, as shown in Figure 1a. The combination of the surfactant (T80) and the co-surfactant (PEG) resulted in the smallest droplet size, whereas SLS resulted in the largest size (Table 1). The zeta-potential is another important parameter that determines the stability of emulsions, as well as their interaction with the biological tissues. The prepared emulsions exhibited zeta-potential ranging from +3.7 ± 0.61 mV to +5.5 ± 0.52 mV, attributed to the presence of chitosan, as shown in Figure 1b and Table 1. The use of a mixed system of surfactants and co-surfactants resulted in a higher positive zeta-potential by keeping chitosan at the surface of the nanodroplets.

#### 3.1.2. Drug Loading and % Entrapment Efficiency

Entrapment efficiency (EE) is an important parameter as it determines the dose and packaging of the emulsions. The percent EE data of the formulated emulsions shown in Table 1 reveals that there was no significant difference (*p* > 0.05) in %EE among F1 (74.3 ± 2.1), F2 (69.7 ± 3.5) and F3 (76.9 ± 2.7). However, the %EE data of the F4 emulsions (80.4 ± 3.2) clearly demonstrates a statistically significant difference when compared with F1, F2 and F3. The %EE data clearly suggests that a combined use of a surfactant and co-surfactant plays a vital role in the enhancement of the %EE of 5-FU. The results of the current investigation are also supported by the study of Artiga-Artigas et al. and Sarheed et al. which reported that the presence of low surface tension between nano droplets covered with a surfactant improves drug solubility and prevents the droplet coalescence to ensure drug retention [37].

#### 3.1.3. Morphology of Emulsions

The morphology has an important influence on the stability of formulations. A microscopic image of the formulated emulsions is shown in Figure 1c. The shape of the nano droplets in the emulsion was found to be spherical. Different compositions of nano formulation did not show any difference in droplet shape.

#### 3.1.4. pH of Emulsions

The acid-base balance plays an important role in development of emulsions as it reflects the suitability of the emulsions on the skin. Table 2 indicates the pH of the formulated emulsions. All formulations showed a pH in the range of 5–6 (that is close to the pH of the skin), which justifies their suitability for topical application [38].

#### 3.1.5. Viscosity of Emulsions

Viscosity plays a key role in the emulsion’s stability and spread ability. The viscosities of all the emulsions were found to be lower than 20 cps (Table 2), except for the F2 formulation that contained SLS. The formulations F1, F2 and F3 exhibited low viscosity values, which is an ideal property of emulsions [39].

#### 3.1.6. Ease of Emulsification and the Phase Separation Study

Transmittance and phase dilution studies were conducted to estimate the emulsification and phase separation of the emulsions. The ease of emulsification indicates the high quality of emulsions as well as the therapeutic efficacy. During the phase separation study, as all the emulsions exhibited no phase separation, they were subjected to an additional assessment. Among the different formulations, F4 showed the maximum transmittance (Table 3), indicating better emulsification properties in comparison to the others.

#### 3.1.7. Skin Irritancy Test

The compatibility of the emulsions was also tested in terms of skin irritancy. The test was performed on the rat ear; the presence of erythema was related to the irritancy potential of the emulsions to the skin. The resultant data given in Table 3 reveal that emulsions F1, F3 and F4 were well tolerated for 24 h, whereas the F2 emulsion exhibited skin irritancy, which is presumed to be due to the presence of SLS in emulsions [40].

#### 3.1.8. Stability Study

**Figure 2:**
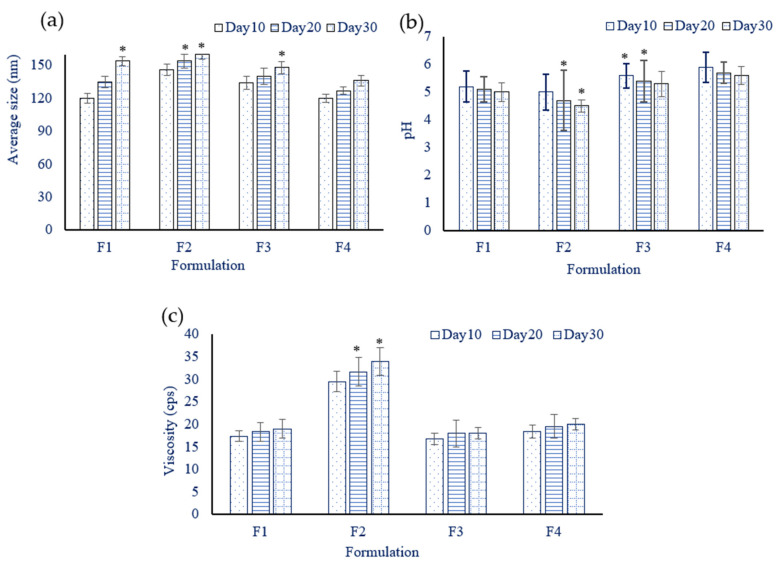
The size of the emulsions containing 5-FU (**a**), variations in the pH of the emulsions containing 5-FU (**b**) and the viscosity of the emulsions containing 5-FU (**c**), data are expressed as mean ± S.D, n = 3, * denotes (*p* < 0.01) statistical significance.

The normality of the distribution of the data by a suitable test such as the Ryan-Joiner (same as Shapiro-Wilk) test or the Kolmogorov–Smirnov (K-S) test was measured before applying a one-way ANOVA. *p* > 0.05 is the probability that the null hypothesis is true. However, a statistically significant test result (*p* ≤ 0.05) means that the test hypothesis is false or should be rejected. A P-value greater than 0.05 means that no effect or changes were observed. The stability of the chitosan-coated emulsions was monitored for 30 days. During this period there was no phase separation, cracking or sedimentation in the emulsions; however, a small variation in droplet size was observed ranging from 10–25 nm in Figure 2a. In size, the normality of the data distribution showed a p-value greater than *p* > 0.15 in the F1 formulation (Day 10, 20), F2 (Day 10), F3 (Day 10, 20), and F4 (Day 10, 20 and 30) and there were no significant effects or changes in size. However, there were significant (*p* < 0.01) changes that occurred in F1 (Day 30), F2 (Day 20 and 30) and F3 (Day 30). It was noticed that the F4 formulation was the most stable among all the nano emulsions in size. This is attributed to its small droplet size which enhanced the stability of emulsions [41,42]. While in storage, the formulations were subjected to pH measurement, as shown in Figure 2b. The data show that were no effects on the pH of formulations F1 and F4 at day 10, 20 and 30 (*p* > 0.15). Moreover, there were significant (*p* < 0.01) changes reported in formulation F2 (Day 20 and 30) and F3 (Day 10 and 20). Usually, the appropriate pH for the topical application of emulsions ranges from 4 to 6. Surprisingly, no significant change (*p* > 0.15) in pH was observed while the formulated emulsions were in storage, which indicates the stability of the formulations. The viscosity of the emulsions was measured during storage (the resultant data is presented in Figure 2c). With regard to the viscosity of formulations F1, F3 and F4 (Day 10, 20 and 30), no significant (*p* > 0.15) changes were reported to have occurred during the stability period, except for the F2 formulation (*p* < 0.01) (Day 20 and 30), which provides supporting data to show the stability of the pharmaceutical formulations. The SLS-containing emulsions exhibited a high viscosity in comparison to the other surfactant-containing emulsions. The durability of the product was determined by observing changes in the droplet size, the pH and the viscosity of the four formulations (F1 to F4). It was observed that no change occurred in any of the three (droplet size, pH and viscosity) parameters in the F4 formulation. Therefore, this study suggests that the F4 formulation is the most stable based on the obtained data.

### 3.2. In Vitro Drug Release Study

**Figure 3:**
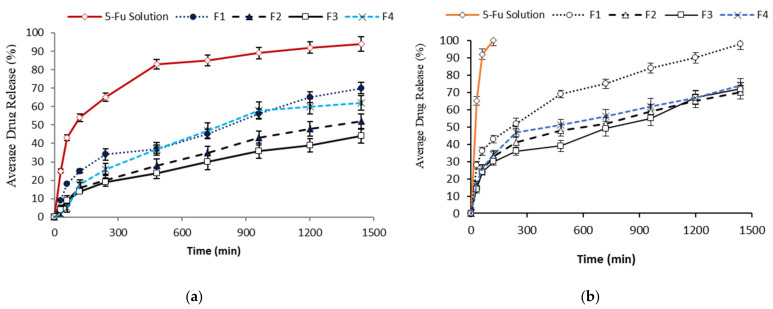
% average drug release of the pure 5-FU solution and the 5-FU emulsion formulations (F1, F2, F3, F4) (**a**), % average drug release of pure 5-FU solution and 5-FU emulsion formulations (F1, F2, F3, F4) using the centrifugation method (**b**), where F4 exhibited significant differences from the rest of the formulations, data are expressed as mean ± S.D, n = 3.

Franz diffusion cells were employed to determine any premature drug release from the developed emulsions on the skin surface. A coating was added to the emulsions using chitosan to control the release of the drug. The drug release of the chitosan coated 5-FU emulsions was compared with uncoated 5-FU emulsions in buffer media at pH 5.5. The chitosan-coated 5-FU emulsions showed significant differences (*p* < 0.05) on the release of 5-FU compared to uncoated 5-FU emulsions. It is noteworthy that the 5-FU release was retarded from all the formulations and less than 35% of 5-FU was released within the first 500 min, whereas more than 80% of 5-FU was released from the solution in Figure 3a. The variation was noticed on the release of 5-FU among the formulations due to the types and concentrations of surfactant used in the formulation. The release of 5-FU from the emulsions was also performed using the centrifugation method. The 5-FU emulsions without a coating were released (100%) within 2 h, whereas the formulation of the emulsions retarded the release of 5-FU. According to the centrifugation method, the release of the drug from the F1 emulsion was significantly higher (*p* < 0.05) than the other formulations. More than 50% of the drug was released between 300 and 600 min, as shown in Figure 3b.

### 3.3. Drug Release Kinetics

The release kinetic mechanism was determined using the Weibull equation shown in Table 4. The value of ‘b’ varied from 0.643 ± 0.54 to 0.897 ± 0.16 and R^2^ ranged from 0.6951 ± 0.47 to 0.8860 ± 0.96. The release kinetic data indicated the diffusion mechanism occurred via Euclidean space (F1), whereas the formulations (F2, F3, F4) also followed a diffusion mechanism within a normal Euclidian substrate, representing a different release mechanism from F1. The drug release mechanism model allows for a better understanding of the delivery system to elucidate the carrier mechanism [43]. It allows the drug release rate to be predicted from the matrix that may provide a preliminary idea to design the formulation.

### 3.4. In Vitro Permeation Studies

**Figure 4:**
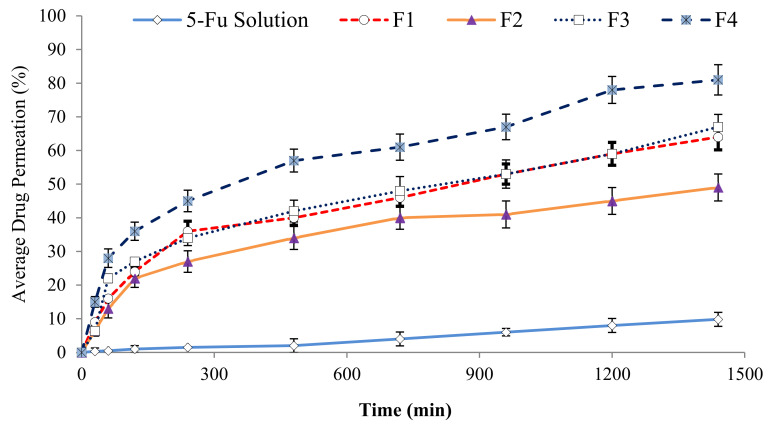
The in-vitro permeation profile of the pure 5-FU solution and the 5-FU emulsion formulations (F1, F2, F3, F4), where F4 exhibited significant difference from rest of formulations, data are expressed as mean ± S.D., n = 3.

Figure 4 shows the amount of 5-FU permeability that occurred across the rat skin in 24 h using the receptor compartment (PBS, pH 7.4) of the Frances diffusion cell. The combination of the surfactants (tween) and the co-surfactant (PEG) showed the best drug penetration profile in the present study. In comparison to the control group (5-FU solution), the formulated emulsions of 5-FU were able to penetrate through the skin. It was found that initially, a higher penetration profile from 0 to 4 h was recorded that gradually reached a plateau due to the small droplet size of the emulsions, the concentration gradient, chitosan, and the surfactants used that directly enhanced the penetration capability through the SC in skin surface [39]. It can be concluded from the results that a reduction in the droplet size increases the permeability of emulsions through the skin. The smaller the droplet size, the better the spread ability covering a large surface area will be, allowing the transportation of the incorporated drugs in the emulsions deeper through the skin [44]. The formulation facilitates the skin diffusion process by the influence of the concentration gradient. The viscosity is another important factor that plays a key role in the penetration of the drug molecules transversely through the skin. Optimum viscosity is needed for the emulsions to pass through the SC; however, emulsions with too low or too high a viscosity will flow down or become sticky. Other factors such as droplet size and high spread ability also contribute to easier penetration across the skin [45]. Increasing the viscosity of the emulsions leads to changes in the formulation type from transdermal to topical drug delivery [46,47]. All formulations coated with chitosan exhibited greater skin permeation of 5-FU. This may be due to the cationic nature of chitosan polysaccharide which interacts with the negatively charged keratin in the skin (lipids and protein) and leads to drug permeation across the skin. However, the SLS-containing formulation showed minimum permeation across the skin, as shown in Figure 4.

This may be due to SLS simply removing the detection layer of lipids above the critical micelle concentration (CMC) [38]. Formulation F3 contained span 20 as a surfactant which may have affected the intercellular lipids by enhancing the fluidity of the emulsion and enhancing diffusivity [39]. Formulations F1 and F4 were incorporated with T80 that caused an increase in the drug solubilization and penetration deeper into the intercellular lipids of the SC due to the non-ionic nature of this surfactant. T80 tends to interact and bind with skin keratin filaments which interrupt the corneocytes. The combination of T80 and PEG 4000 in F4 exhibited the highest permeation across the skin, as shown in Figure 4. The surfactants may have a dual effect in enhancing drug permeation on the skin components. PEG 4000 also acts as a drug permeation enhancer because of its potential interaction with the lipid constituents of the skin layer. The diverse effects of the surfactants on drug infiltration depends entirely on their ability to disrupt or fluidize of lipid composition of the SC [44].

### 3.5. Skin Drug Retention

**Figure 5:**
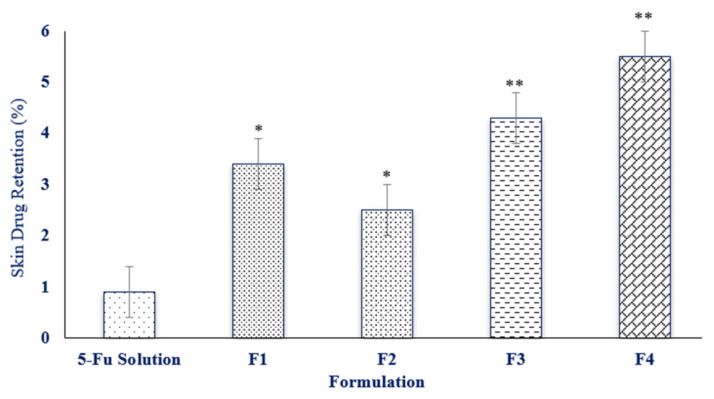
The in vitro skin drug retention profile of the pure 5-FU solution and the 5-FU emulsion formulations (F1, F2, F3, F4), where F4 exhibited significant differences from the rest of formulations, data are expressed as mean ± S.D, n = 3, * denotes (*p* ˂ 0.05) and ** denotes (*p* ˂ 0.01) statistical significance.

Figure 5 depicts the amount of drug retained within the skin after 24 h. The 5-FU concentrations in the skin were significantly higher in the emulsions in comparison to the simple drug dissolved in the PBS buffer alone after an interval of 24 h (*p* ≤ 0.05). Formulation F4 had the maximum skin drug retention in comparison to the other formulations. Hence, F4 can be considered as a potential topical formulation because of its cutaneous retention of the drug within the skin. The small droplet size and the synergistic effect of combination of surfactants could be the potential factors for drug accumulation on the skin [41]. The results revealed that the difference in particle sizes also influences the drug uptake within the skin layers. The exact mechanism that causes such an increased drug uptake is still unclear and further investigations must be performed in the future.

### 3.6. The Physicochemical Characterization of the Skin

**Figure 6:**
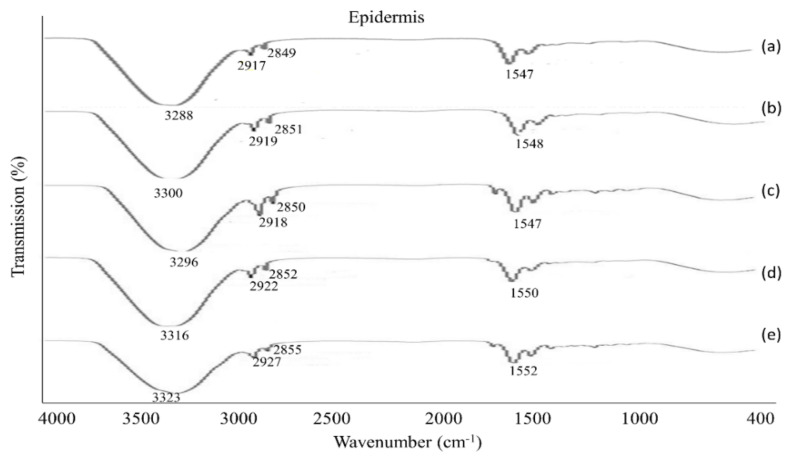
ATR-FTIR spectra of the epidermis (**a**) untreated skin, and skin treated with (**b**) F1, (**c**) F2, (**d**) F3 and (**e**) F4., data are express as mean ± S.D, n = 3.

After the permeation study, the tested skin was employed for ATR-FTIR analysis to determine the mechanism of the drug permeation across the skin membrane. The ATR-FTIR data reveal that all the formulations were influential in disturbing or fluidizing the lipids and proteins of the skin, except formulation F2. The fluidization of skin lipids and proteins intensified the permeation and retention of drugs in the skin. The peaks appeared in the epidermis at 3300 cm^−1^, 2920 cm^−1^ and 2850 cm^−1^ that moved to a high frequency in the emulsion samples (F1, F3, F4), as shown in Figure 6.

**Figure 7:**
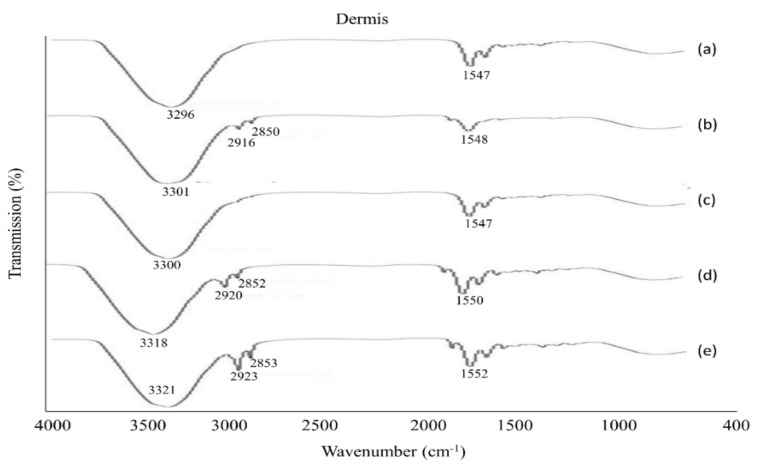
ATR-FTIR spectra of the dermis (**a**) untreated skin, and skin treated with (**b**) F1, (**c**) F2, (**d**) F3 and (**e**) F4, data are expressed as mean ± S.D., n = 3.

The peak that appeared from 3300 cm^−1^ to 3330 cm^−1^ represents O-H and N-H groups of keratin, ceramid, and additional lipophilic components in the SC [18,37]. This implies that emulsions primarily affect the lipids and proteins of the SC resulting in a high level of drug permeation across the skin. In the dermis, the spectra data showed the fluidized nature of the skin where peaks appeared from 3280 cm^−1^ to 3330 cm^−1^, corresponding to the presence of O-H and N-H groups [47]. The peaks that appeared from 2850 cm^−1^ to 2930 cm^−1^ signify the asymmetric groups (CH_2_), of skin keratin, lipids, and ceramide in formulations (F1, F3, F4), shown in Figure 7.

## 4. Conclusions

The current study intended to develop modified chitosan-coated emulsions containing 5-fluorouracil for transdermal delivery. The incorporation of olive oil, and a combination of surfactants and chitosan-coated 5-FU emulsions exhibited a uniform droplet size, representing the potential stability of the emulsions within an acceptable range of pH, suitably controlled drug release, increased skin permeability and a deeper penetration across the skin. The drug permeation data suggested that formulation F4 exhibited the highest permeation across the skin up to 1500 min. This could be attributed to the combination of T80 and PEG 4000 that can overcome the obstacle of drug solubilization and penetration via an interaction with and binding with skin keratin filaments. The formulated modified chitosan-based emulsions of 5-FU developed in this study can be considered as promising potential topical carriers for the controlled-release delivery of 5-FU due to its cutaneous retention of the drug within the skin. The emulsions of 5-FU developed in this study are not only expected to offer better drug delivery in comparison to conventional drug therapies, but they are also predicted to be able to overcome the dosing-related side effects and toxicity. However, further optimization studies including stabilization and targeting should be performed both in vitro and in vivo. The current study recommends that formulated emulsions of 5-FU should be investigated for further clinical studies.

## Figures and Tables

**Table 1:** Results of the size, PDI and zeta potential of the emulsions.

F. Code	Average Size (nm)	Polydispersity Index	Zeta Potential (mV)
F1	120.1 ± 10.52	0.281 ± 0.04	+3.9 ± 0.73
F2	141.3 ± 9.31	0.326 ± 0.08	+3.7 ± 0.61
F3	129.5 ± 8.92	0.272 ± 0.04	+4.6 ± 0.66
F4	109.6 ± 7.23	0.241 ± 0.03	+5.5 ± 0.52

Data were expressed as mean ± S.D., n = 3.

**Table 2:** pH and viscosity of the formulated emulsions.

F. Code	pH	Viscosity (cps)	Drug Content	%EE
F1	5.2	17.3 ± 1.32	88.5 ± 3.4	74.3 ± 2.1
F2	5.0	29.5 ± 1.22	86.9 ± 3.3	69.7 ± 3.5
F3	5.6	16.7 ± 1.13	90.7 ± 3.6	76.9 ± 2.7
F4	5.9	18.4 ± 2.09	92.1 ± 2.9	80.4 ± 3.2

Data were expressed as mean ± S.D., n = 3.

**Table 3:** Ease of emulsification, phase separation, skin irritation and stability of the emulsions.

F. Code	No. of Flask Inversions	% Transmittance	Phase Separation	Skin Irritancy	Stability
F1	5	85.53	No	No	Stable
F2	7	79.20	No	Yes	Stable
F3	6	87.91	No	No	Stable
F4	4	97.7	No	No	Stable

**Table 4:** The release kinetic mechanism using the Weibull equation.

F. Code	b	R^2^
F1	0.643 ± 0.54	0.695 ± 0.47
F2	0.850 ± 0.43	0.755 ± 0.42
F3	0.767 ± 0.79	0.789 ± 0.92
F4	0.897 ± 0.16	0.886 ± 0.96

Data are expressed as mean ± S.D., n = 3.

## Data Availability

The data presented in this study are available on request from the corresponding author.
